# Supplementary figures and images for: A drug repurposing screen for whipworms informed by comparative genomics
Source: PLoS Negl Trop Dis. 2023 Sep 5;17(9):e0011205. doi: 10.1371/journal.pntd.0011205 (PMC10503962; doi:10.1371/journal.pntd.0011205)

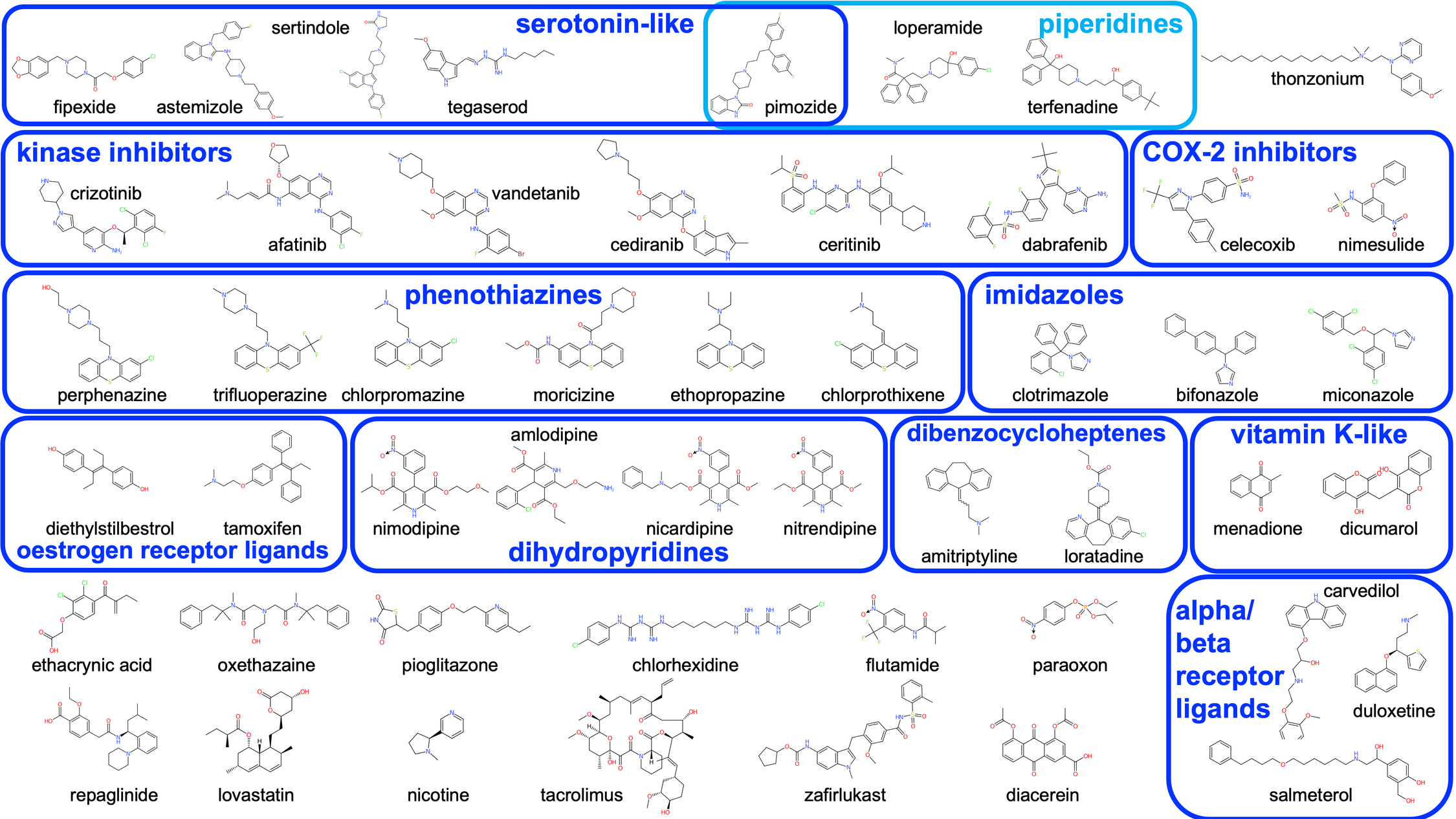

Supplement: S1 Fig — The images of compounds were generated using the CDKDepict website [45]. The salt form tested is given in S3 Table. (TIF) [file pntd.0011205.s007.tif]

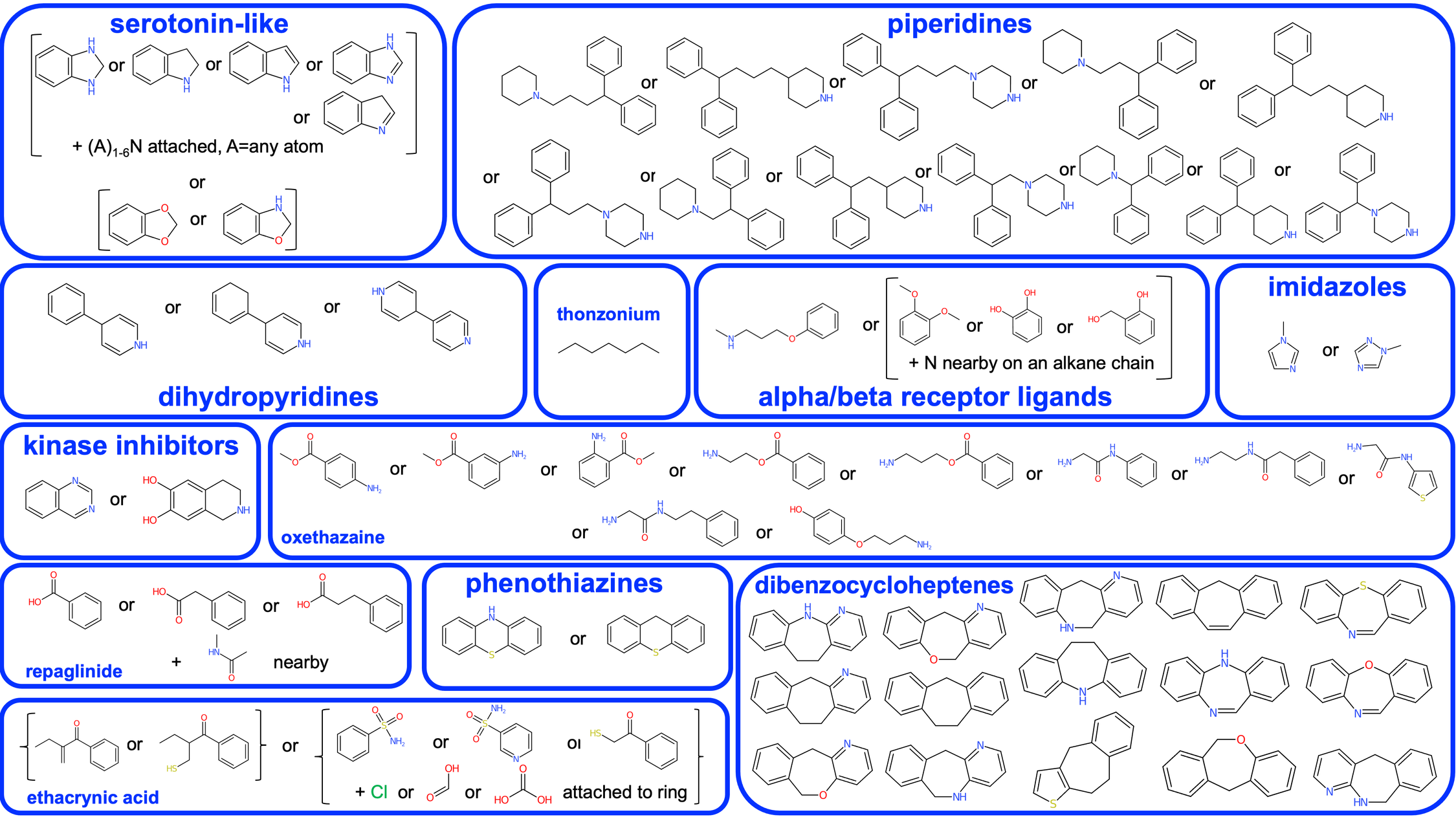

Supplement: S2 Fig — The substructures that were used in the ‘substructure search function’ in DataWarrior, to search for additional approved drugs with substructures present in our top 50 hits. Images of compounds were generated using the CDKDepict website [45]. (TIF) [file pntd.0011205.s008.tif]

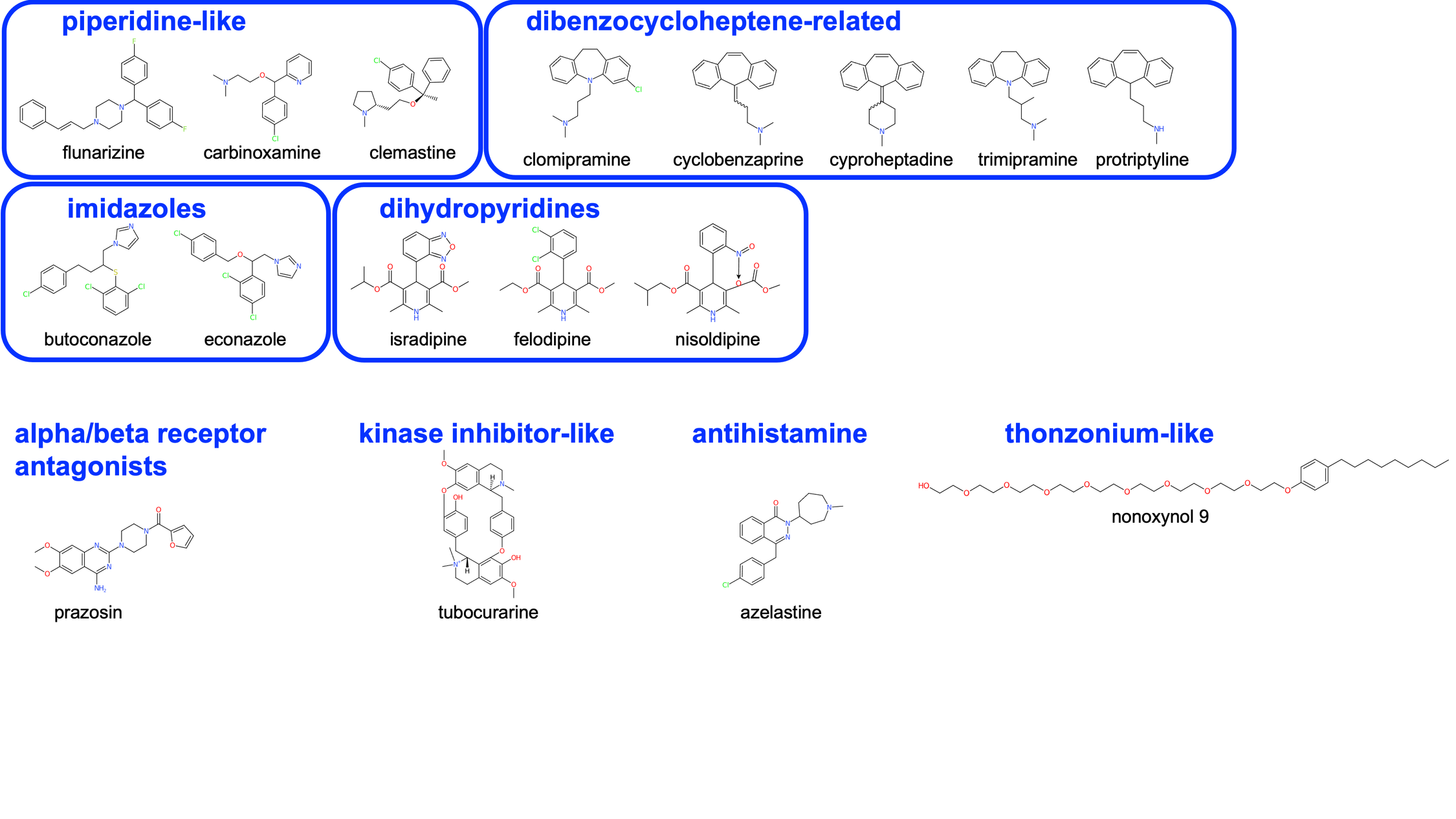

Supplement: S3 Fig — The images of compounds were generated using the CDKDepict website [45]. The salt form tested is given in S4 Table. (TIF) [file pntd.0011205.s009.tif]

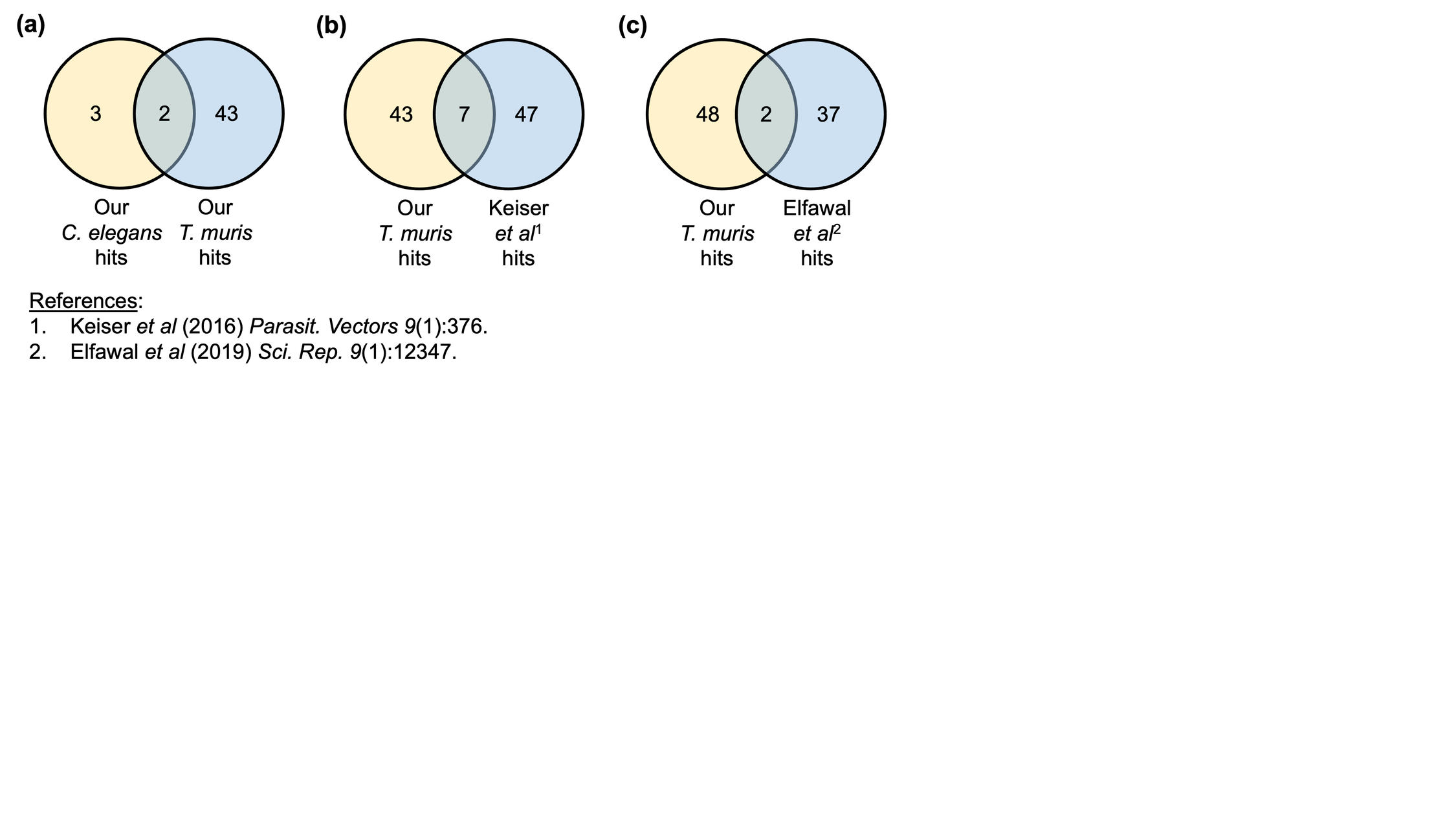

Supplement: S4 Fig — (a) Overlap between our hits in C. elegans and in T. muris, after re-screening at 100 μM, (b) overlap between our hits in T. muris, and those of Keiser et al 2016 [23] in Ancylostoma ceylanicum, (c) overlap between our hits in T. muris, and those of Elfawal et al 2019 [29] in A. ceylanicum. (TIF) [file pntd.0011205.s010.tif]

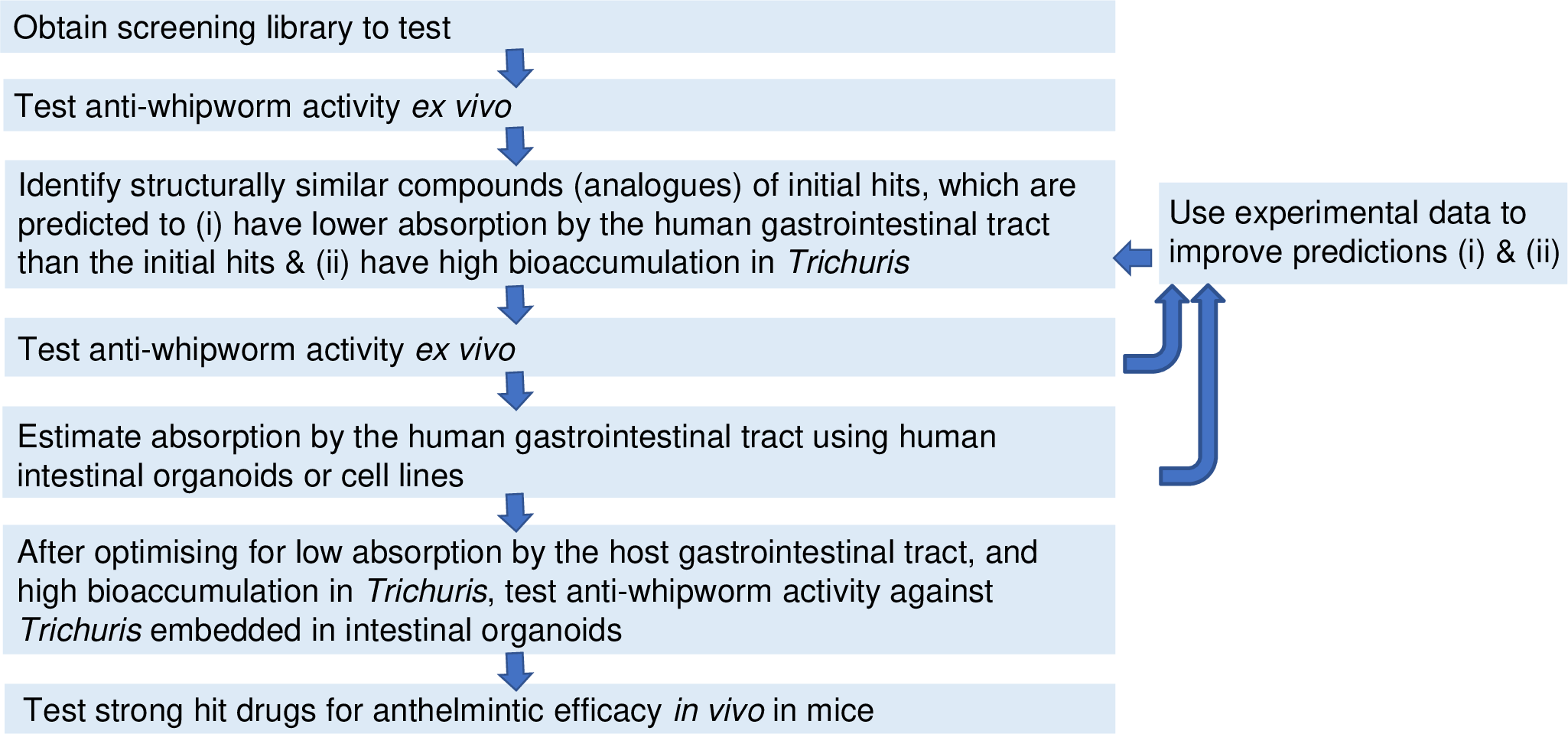

Supplement: S5 Fig — (TIF) [file pntd.0011205.s011.tif]
