# Supplementary material for: A drug repurposing screen for whipworms informed by comparative genomics
Source: PLoS Negl Trop Dis. 2023 Sep 5;17(9):e0011205. doi: 10.1371/journal.pntd.0011205 (PMC10503962; doi:10.1371/journal.pntd.0011205)
Supplement: Attachment — Submitted filename: ReviewersComments_21June2023alc.docx [file pntd.0011205.s012.docx]

**Results**

Reviewer #1: Unless I am missing it, there doesn’t appear to be a figure on the C. elegans ex vivo drug screen? These data are interesting and the paper would benefit from adding a figure summarizing these results. This result seems consistent with a previously report for ex vivo hookworm screen (PMID: 31451730) where C. elegans had limited overlap.

We had 14 initial hits in *C. elegans*, and 5 were statistically significant (*P*<0.05) after re-screening at 100 µM (ponatinib, chlorhexidine, clotrimazole, sorafenib, and paroxetine, as listed in the Results section); we had 50 initial hits in *T. muris*, 45 were statistically significant (*P*<0.05) after re-screening at 100 µM (listed in Supplementary Table 3); and only 2 hits were statistically significant in both *C. elegans* and *T. muris* after re-screening at 100 µM (clotrimazole and chlorhexidine, as listed in the Results section). A Venn diagram summarising these results has now been included as S4 Fig (cited on line 361):

**Editorial and Data Presentation Modifications?**

Reviewer #1: Minor points:

1. Is motility an antiparasitic mechanism for drugs acting on whipworms, or is the goal to actually kill the worm? If paralysis is not necessarily a sufficient outcome (since the anterior of the worm is embedded in the intestinal mucosa and so the worm may not wash away), it may be useful to reassay motility after drug washout. I bring this up not as a request for more data, but a possible discussion point. But if the authors are aware of whether their ex vivo hits such as aminergic compounds were actually killing or just paralyzing the worms, this would be interesting to comment on.

Our screening platform INVAPP is designed to detect reductions in motility [Partridge et al 2018 PMID:29223747], so cannot distinguish whether a worm has been killed or simply paralysed by a drug. However, several known anthelmintic drugs for soil-transmitted nematodes such as levamisole, pyrantel, oxantel, and piperazine, act by paralysing the worms, thereby allowing the host body to easily expel the parasites [Martin et al 2015 PMID:24871674]. Although the benzimidazoles albendazole and mebendazole are the most widely used drugs for trichuriasis, pyrantel and levamisole have also been used for treating trichuriasis [Keiser et al 2013 PMID:23556013].

We have added a sentence to the Introduction (line 126), when we introduce INVAPP, saying: “*This is an attractive approach since several drugs that have been used in the past to treat trichuriasis, such as levamisole, pyrantel, and oxantel [Keiser et al 2013 PMID:23556013], cause spastic paralysis of the worms, thereby facilitating their expulsion (while still alive) by peristalsis of the host intestine [Martin et al 2015 PMID:24871674].*”

2. The authors note that in compound selection they made a choice to discard annotated antipsychotics. I wonder if this is a decision that should be made during compound selection because several hits seem to either be antipsychotics or structurally very similar to antipsychotics. Terfenadine is similar in structure to antipsychotic haloperidol and numerous tricyclic antidepressants are still in the list (ex. chlorprothixene, and cyproheptadine is closely related to TCAs). Pimozide which was a hit and tested in mice and is an antipsychotic. Is this still a triage step that should be used going forward?

We included antipsychotic and antidepressant drugs in our initial screen, because we hoped that if we identified an antipsychotic/antidepressant drug as a hit, we could then find a structurally similar approved drug that was not antipsychotic/antidepressant (e.g. an antihistamine) to test against *Trichuris*.

For clarification, we have added a sentence to the Results paragraph 1 (line 275): “*Some of the drugs tested (e.g. antipsychotics) may be unsuitable for repurposing, but were included in the hope that if they were hits, we would next identify structurally similar, but safer, approved drugs (e.g. antihistamines) to test*”.

While pimozide is an antipsychotic, it has also been used for non-psychotic disorders such as tics in Tourette’s syndrome [Pringsheim 2009 PMID:19370666], and since it had the highest activity in our *ex vivo* screen, we tested it in mice in the hope that a low dose could reduce worm burden without serious side effects.

For clarification, we’ve added a sentence to the Results (line 391): “*Although pimozide is an antipsychotic, it has also been used for non-psychotic disorders such as tics [Pringsheim 2009 PMID:19370666], so we tested it in mice in the hope that a low dose could reduce worm burden without serious side effects*.”

3. Perhaps in the legend of somewhere the putative nematode targets of ex vivo hit compounds could be elaborated on? This may be in the 2019 paper, but I was wondering how antifungals (econazole, butoconazole) or surfactants / detergents (nonoxynol-9, thonzonium) may be acting on worms.

In Elfawal *et al* 2019 [PMID:31451730], the imidazole antifungals econazole and sulconazole have activity against hookworms and are predicted to target nematode cytochrome CYP-450, since CYP-450 is the target of imidazoles in fungi and the target of the imidazole miconazole in the flatworm *Schistosoma mansoni* [Ziniel 2015 PMID:26713732]. In contrast, another imidazole, DII (diisopropylphenyl-imidazole), was shown to have activity against *C. elegans*, by targeting *unc-29*, a constituent of a muscle-expressed nicotinic acetylcholine receptor [Blanco 2018 PMID:30557347]. With respect to the surfactants thonzonium and nonyxnol, they disrupt the cell membranes of bacterial or sperm cells, respectively, so may disrupt the cell membranes of nematode epidermal cells.

We have added the following subscript to Table 1 (line 318): “*The target of the imidazoles in nematodes may be the cytochrome CYP-450 [Elfawal et al 2019 PMID:31451730] and/or a nicotinic acetylcholine receptor [Blanco 2018 PMID:30557347]; while the surfactants thonzonium, nonoxynol, and chlorhexidine may disrupt the cell membranes of nematode epidermal cells*.”

4. The authors refer to high hit rate in primary screen (12%) supporting utility of a comparative genomics approach to compound selection. A question in my mind when reading was to what degree this improved over a baseline for a typical screen. Perhaps worth expanding upon by citing references of other whipworm screens (understanding that screening methodology may of course vary)?

The fraction of hits in a screen depends on the life cycle stage studied, screening library, the drug concentration tested, the screening platform, and threshold used to define a hit. Using the INVAPP screening platform [Partridge et al 2018 PMID:29223747], a library of 480 drug-like small molecules was previously screened at 100 µM against *Trichuris muris* adults, resulting in 20 primary hits (4.2% hit rate) [Partridge et al 2017 PMID:28182663]. Using a different screening platform, Cowan *et al* 2016 screened a library of 114 approved human oncology drugs at 50 µM against *T. muris* L1 larvae, and found 12 hits (10.5% hit rate). Therefore, the rate of hits for a library of drug-like small molecules may be relatively low (<5%), while the rate of hits for a library of approved drugs that are known to include many with targets homologous to *Trichuris* proteins (as in the case of our study and the Cowan *et al* study of human oncology drugs) may be significantly higher (>10%).

For clarification, we’ve edited the first paragraph of the Discussion (line 435) to say “*Our hit rate of ~12% (50/409) was relatively high compared to a previous rate of ~4% (20/480) for a library of drug-like small molecules screened against* T. muris *using INVAPP [Partridge et al 2017 PMID:28182663]. Thus, this demonstrated the utility of our comparative genomics approach to predict parasite proteins for targeting by approved drugs. Similarly, a screen of human oncology drugs against T. muris by Cowan et al 2016 [PMID:27498843] also found a relatively high hit rate (10.5%), presumably because T. muris has homologues of many of the human targets.”*

5. Would optimizing for low absorption across the host GI tract also impair uptake across nematode tissues? Would more polar, less lipophilic compounds decrease diffusion into nematode?

We agree that optimising for low absorption across the host GI tract may also impair uptake across nematode tissues. Although it is not a simple relationship (see Wils *et al* 1994 PMID:8182532), more polar, less lipophilic compounds may be less likely to be absorbed by the host GI tract and also less likely to enter the nematode. Indeed, Burns *et al* 2010 [PMID:20512140] used HPLC to perform a survey of accumulation and metabolism of >1000 compounds in *C. elegans* and found that a key predictor of bioaccumulation in *C. elegans* is the lack of any of five chemical fragments. Three out of these five fragments were hydrophilic moieties (e.g. a carboxylic acid group, an aliphatic hydroxyl group or a sulfonyl group) that (i) likely reduce solubility in aqueous buffer and so cell membrane permeability, and that (ii) also are known to be sites of phase II conjugation reactions in mammals (e.g. glucuronidation, glycosylation, and sulfation), which likely leads to modification and excretion by the worm. On the other hand, compounds that accumulated in *C. elegans* generally lacked these fragments and had a high LogP (i.e. were non-polar, hydrophobic compounds). Based on their findings, Burns *et al* 2010 [PMID:20512140] developed a predictive (machine learning) model for bioaccumulation in *C. elegans*. This model may also be useful for predicting bioaccumulation in *Trichuris*. We also would hope that as we gather more experimental data on which compounds are active against *Trichuris ex vivo*, in organoid models, and in mouse models, we will be able to produce better predictive models for bioaccumulation in *T. muris* and for absorption by the mammalian gastrointestinal tract, to help us identify more compounds that like mebendazole are taken up well by the worm but not by the human gastrointestinal tract.

To highlight this issue, we have added the following sentence to the last paragraph of the Discussion (line 508): “*While doing so, it would be important to avoid adding chemical fragments predictive of low bioaccumulation in nematodes (identified by Burns et al 2010 [PMID 20512140] for C. elegans), as our goal is to identify compounds that like mebendazole are taken up well by the worm but not by the human gastrointestinal tract.*”

**Summary and General Comments**

Reviewer #1: There is a need for new anthelmintics to treat whipworm infections, given that drugs such as mebendazole can be subcurative. This manuscript reports the results of a medium throughput drug screen on T. muris, with the aim of identifying new therapies or lead anthelmintic compounds. Specifically, authors chose these based on comparative genomics approach focusing on small molecules likely to act on key whipworm targets. The data are clearly presented and the rational for the workflow of the screen progressing from ex vivo primary screen to testing potency to dosing mice in vivo is clearly articulated. While none of the ex vivo hits displayed in vivo efficacy, these data are still quite useful to the community and will inform the design of future screening efforts. The authors propose reasonable explanation for the discrepancy between the ex vivo and in vivo results, noting that many compounds optimized for high host bioavailability (uptake from GI) when in fact
whipworm may require opposite if diffusion across posterior of animal is main route of parasite exposure. The main area I would like to see some more elaboration on in the results is the C. elegans screening data.

We have now added a Venn diagram showing the number of shared hits between *C. elegans* and *T. muris* (see above). We have also added some sentences to the fourth paragraph of the Results (line 361):

“*Clotrimazole and other imidazoles may have cytochrome CYP-450 as a conserved target across fungi, flatworms, and nematodes [Elfawal et al 2019 PMID:31451730; Ziniel 2015 PMID:26713732], or alternatively may target a nicotinic acetylcholine receptor in nematodes [Blanco 2018 PMID:30557347]. Chlorhexidine, a surfactant, may disrupt the cell membranes of nematode epidermal cells, as it does for bacterial cells*.”

Reviewer #2: The Coghlan et al manuscript is a very quick, easily absorbed manuscript that I found to be insightful, especially in the discussion’s commentary. I do think that PNTD is an appropriate venue for its publication.  I have only minor suggestions for improvement.
1. I am not sure the last sentence in the abstract is appropriate for that section (too prescriptive for an abstract?) but I leave that to the authors to consider further.

We agree and have removed that sentence **(**“*Therefore, we recommend that prior to in vivo studies, future researchers first assess drug absorption by the host, for example, using human intestinal organoids or cell lines, and drug uptake by whipworms using intestinal organoids infected with T. muris*”.)

2. Table 1, column G has some nice citations showing previous work on the indicated compounds against helminths. However, I would also be interested to see the overlap (and non-overlap) of molecules screened and hits obtained in the Venn diagram with the authors current work and that of Keiser et al 2016 and Elfawal et al., 2019 (PMID 31451730).

Keiser *et al* 2016 [PMID:27363703] and Elfawal *et al* 2019 [PMID: 31451730] did not provide the names/structures of the sets of compounds that they screened, so we were not able to compare the set of 409 approved drugs that we screened to the complete sets of 1600 and 1280 approved drugs in their respective papers. However, we have compared the 50 hits that we found in our screen against *Trichuris muris* adults at 100 µM to the 54 hits found by Keiser *et al* 2016 against *Ancylostoma ceylanicum* L3s at 200 µM, and to the 39 hits found by Elfawal *et al* 2019 against *Ancylostoma ceylanicum* adults at 30 µM. This comparison is shown in the following Venn diagrams, which we have added as a Supplementary figure 4B and 4C:

We also added a sentence saying (line 468):

*“For example, 7 of the 50 hits were previously reported in a screen against* Ancylostoma ceylanicum *L3s at 200 µM by Keiser et al 2016 [PMID:27363703], while 2 of the 50 hits were previously reported in a screen against* A. ceylanicum *adults at 30 µM by Elfawal et al 2019 [PMID:31451730] (Table 1; Supplementary Figure 4B, 4C).”*

3. It would be convenient to interested readers to have a supplementary table (or modify Sup Table 1) that has all examined molecules (409+71) and their in vitro results, whether or not they are considered ‘hits’, and their in vivo results, all in different columns. This helps for others to analyzed the data for comparison purposes, although all of the releveant data is present, but in different files and slightly different formats etc.

While we understand and appreciate the reviewer’s point, merging all data into one large supplementary table would make it difficult for many readers to navigate and see the key results at a glance because different sets of variables were used for different subsets of data (e.g. mean motility at 100 µM in the initial screen for 409 compounds in Supp. Table 1; *P*-values for re-screening for 50 compounds at 100, 20 and 50 µM in Supp. Table 2; mean motility at 100 µM for 71 additional compounds and *P*-values for re-screening them at 100 and 50 µM in Supp. Table 3; EC_50_ for 14 compounds in Suppl. Table 4; worm burden reductions in mice for 9 compounds in Table 2). We have tried to make the individual tables easy to parse and read to increase the re-usability of the data. Therefore, while we thank the reviewer for his/her suggestion, we prefer to keep the tables as they are.

4. It might be useful to the read to have a few more sentences in the introduction that describes in more detail how the 817 compounds were identified, and how the 409 molecules were selected as a subset.

We have added the following additional text to the Introduction (line 116):

“*Briefly, we prioritised worm proteins that had high similarity to their top BLASTP match in ChEMBL, that lacked human homologues, and had* Caenorhabditis elegans/Drosophila melanogaster *homologues with lethal phenotypes [International Helminths Genome Consortium 2019 PMID:30397333]. For each worm protein, we retrieved compounds with high potency/affinity for its top ChEMBL target. After collapsing compounds within a single chemical class to one representative, the resultant set included 817 approved drugs, of which we purchased as many as possible within our budget (prioritising the cheapest), i.e. 409 compounds.*”

5. Another good reference for the ADME properties in elegans to go along with Lindblom and Dodd 2006 in the discussion is the Burns et al 2010 Nat Chem Bio paper (PMID 20512140).

We thank the reviewer for this suggestion. The additional reference has been added to the Discussion (line 453).

6. I found the last paragraph of the discussion of particular interest- perhaps it deserves its own pipeline figure?

We have added a new pipeline figure to go with the last paragraph of the Discussion, as Supplementary figure 5 (cited on line 508):

While I do like the ideas, and they deserve articulation, I do think the authors should consider additional issues that go along with the suggested pipeline. The great thing about screening for established drugs for off-labeled use is that everyone, in principle, is behind that. The drug companies potentially make more money with expanded use, and there can be fast-track to the clinic.  However, when folks start analoging, there may be patent issues to be dealt with- their will likely be infringement against the patents of the original drug (because they are typically broad), or if the patents have run out, then the analog is in the public domain and industry has no incentive to develop the compound further. A second issue is that there may be no fast track to the clinic with an analog of a well characterized and approved drug.
If the authors can envision routes around these issues, they should articulate those routes. If the authors can’t envision ways around these issues, they should discuss the limitations of bringing an effective molecule to market.  Regardless, I find it all very interesting either way.

The reviewer is correct that our suggestions for future screens in the last paragraph of the Discussion depart from using the repurposing strategy. We have added a sentence to the last paragraph of the Discussion to clarify this (line 522):

*“This analogue-based strategy loses the ‘fast-track to the clinic’ benefit of direct repurposing, but may be necessary for trichiuriasis because most approved drugs have been optimised for human bioavailability and are therefore rapidly absorbed by the host gastrointestinal tract.”*

The reviewer has interpreted our suggestions in the Discussion for future screens to just apply to screens of approved drugs, but we had intended them to also be for screens of other (non-approved) compounds. To make this clearer, we have altered a sentence in the Discussion to say (line 505):

“*We suggest that one strategy would be to take the initial hits from a screen (of approved drugs and/or of other compounds) as leads, and then identify structural analogues that are predicted to have lower absorption (compared to the lead compounds) by the human gastrointestinal tract*”.

With respect to the patent issues regarding an analogue of an approved drug, yes, it is correct that analogues will have different patent scope and will likely lack a fast-track to the clinic.

7. Minor note- Figure 1, fourth blue box, I think ‘too’ can simply be removed.

This has been done as suggested.

8. At some point in the future (but beyond the scope of this manuscript), it might of interest to the authors to test the nematode effective analog, nemadipine, in vitro and in vivo given the the overall effectiveness of the DHPs.

Yes, nemadipine-A would be a very interesting one to test against *Trichuris*. It was originally shown to have activity against *C. elegans* [Kwok et al 2016 PMID:16672971] but as far as we know it has not been tested against *Trichuris*.

Reviewer #3: Review of Avril Coghlan et al
“A drug repurposing screen for whipworms informed by comparative genomics”

The manuscript is interesting, helpful, informative, well-written, and easy to read. In a previous report, using comparative genomic tools, the authors identified 409 drugs approved for human use predicted to target essential parasitic worm proteins based on homology to known druggable targets in other species. In this report, the authors tested the possibility of repurposing these 409 compounds towards parasitic nematodes using initial ex vivo screening, dose-response, and in vivo testing. The paper addresses a critical issue in drug discovery against parasitic nematodes: the disagreement between ex vivo potency and in vivo efficacy. Even though the investigators identified 14 compounds with ex vivo potency, none showed significant in vivo activity when tested in mice infected with the murine whipworm parasites. The authors give a detailed discussion on the reason behind such discrepancy and offer suggestions to modify the whipworm screening pipeline to better predict in
vivo activities in mice.
The paper is suitable for publication after significant revision addressing and commenting on the following issues.
Introduction:
The introduction is informative but relatively light. It may benefit the general readers if the authors address the challenges facing drug discovery in parasitic nematodes, including, but not limited to, the lack of culture systems in parasites and the expectation/requirement that final candidates must show reasonable activity against multiple species of human gastrointestinal nematodes.

We thank the reviewer for the suggestion. We have added some lines to the Introduction to explain why drug discovery in parasitic nematodes is challenging (line 94):

***“****Drug discovery for trichuriasis and other parasitic nematodes is challenging given the absence of ex vivo culture systems. Caecal organoids are a promising platform for in vitro/ex vivo culture of whipworms [Duque-Correa et al 2022 PMID:35365634], but are still in the early stages of development. On the other hand, parasite maintenance in mice is expensive, labour-intensive, and has ethical costs. In addition, since there is also a great need for new treatments for other soil-transmitted nematodes (e.g. Ascaris, hookworms), ideally one wants compounds effective against several parasitic species. Furthermore, even if promising hits are found in a screen, because soil-transmitted nematodes affect low income countries, there are financial challenges for pharmaceutical companies to bring an effective molecule to market, which requires years of investment to characterise its pharmacology and safety [Partridge et al 2020 PMID:32550933].”*

It is worth noting why the authors, among all STH, focused their screening against whipworm parasites, as new drugs are equally urgently needed to replace benzimidazoles due to the spreading resistance.

We have added sentences to the Introduction to explain why we focussed on *Trichuris* in our drug screen, and to highlight evidence of benzimidazole resistance in Trichuris in humans (line 91):

*“Indeed, of the three major soil-transmitted helminthiases, current drugs have the lowest efficacy for trichuriasis [Moser et al 2017 PMID:28947636, Partridge et al 2020 PMID:32550933]. Furthermore, there is worrying evidence of resistance to mebendazole and albendazole in* Trichuris *in humans [de Oliveira et al 2022 PMID:35895340, Grau-Pujol et al 2022 PMID:35895348].”*

Methods:
The methods section needs careful revision as many of the listed assays need more essential details. The initial screen at 100µM is relatively high, and the authors need to comment on how they determined the screening dose.

We previously found that 100µM is a good concentration for an initial screen in *Trichuris* using our INVAPP screening platform, and that any hits can be confirmed at lower concentrations in a secondary screen (e.g. see Partridge et al 2017 PMID:28182663). To clarify this, we added a sentence to the Methods (line 189):

*“We previously found that 100µM is a good concentration for an initial screen against Trichuris using our INVAPP screening platform [Partridge et al 2017 PMID:28182663].”*

C. elegans is a free-living nematode, and it is unsuitable to call the assay Ex vivo, as the term is more specific to tissues or parasites tested outside the host body.

We agree with the reviewer and have now changed the heading in the Methods (line 197) from ‘*Ex vivo Caenorhabditis elegans* drug screen’ to ‘*Caenorhabditis elegans* drug screen’.

So far, thousands of strains have been generated with multiple wildtypes isolates already available in C. elegans. The authors need to give more information on the strain of C. elegans they used.

We used *C. elegans* N2. We have added this to the Methods in the sentences (line 197):

“*To obtain C. elegans strain N2 worms for screening, synchronised L1 worms were prepared as described in (Partridge et al. 2018). C. elegans* var Bristol*, originally isolated from mushroom compost near Bristol in the UK, is referred to as N2.*”.

How many L1 were tested per replicate?

We tested approximately 20 L1s per replicate. We have added this to the Methods in the sentence (line 199):

“*In the screen, worms were cultured at 20°C for 6 days in 96-well plates containing E. coli food, with approximately 20 L1s per well, and 100 µM of tested drug (1% v/v DMSO) or a diluted DMSO control, as above*”.

6 days of L1 assay is quite long when incubated at 20c for two reasons. First, at this temperature, for example, in the N2 strain take about three days to develop into adults-again, depending on the strain used. If a single L1 survives, the treatment will eventually grow into an adult and reproduce many F1s. which could lead to a higher false negative rate, especially if the readout is cumulative motility.

Second, six days assay could result in an increased rate of drug metabolism. For example, suppose a specific drug induces paralysis or a reversible kind of activity. In that
case, the treated worms will gain viability after metabolizing the drug, leading to a higher rate of false negatives, and scoring the assay plates when the control wells at the early L4 stage, about 44 hours, could be better here.

This length of assay was similar (six versus five days) to that used in Partridge et al 2018 [PMID:29223747] and was selected to be slightly longer than one L1-L1 life cycle in our 96-well liquid culture assay at 20 °C and to give robust movement scores. We agree with the reviewer’s comment that this could potentially lead to false negatives due to partially penetrant effects or drug metabolism. We would suggest that an advantage is that having the whole developmental cycle in the presence of compound enabled us to test for effects on more biological/developmental processes. We have therefore added discussion of the reviewer’s important points to the Discussion (line 453) as follows:

*“A limitation of our comparison between the T. muris and C. elegans screens is the longer compound exposure time used (C. elegans grown for six days from the L1 stage in the presence of compound at 20 °C before measurement). This may have led to false negatives in the C. elegans compound screen due to metabolism of an active compound or incomplete compound effect being followed by recovered C. elegans growth. Future work should investigate how the design of C. elegans screens could be optimised to model more closely the drug exposure time and worm biological activity of an in vivo parasitic nematode treatment assay and thus hopefully improve the predictive power of C. elegans assays.”*

Assays with multiple replicates are very useful when motility is the only readout, as the range of motility in whipworms is quite large, even in the control group. For example, male whipworms are much smaller and generate lower pixel change than females. When testing the 71 additional approved drugs for prioritization, the authors tested four in total worms, one worm at 100µM, two worms at 50µM, and one worm at 20µM. Single or double-worm motility readout will not generate useful or reliable information. The lack of reasonable replication gives the impression that the 17 compounds selected for retesting at 100µM were chosen arbitrarily.

For the 71 additional approved drugs tested *ex vivo*, we had a limited number of mice available from our in-house *Trichuris* life cycle, and so a limited number of worms (extracted from mice) available. We therefore conducted an initial screen to prioritise these compounds for testing, using four replicates (one worm at 100µM, two worms at 50µM, and one worm at 20µM). This experiment was not powered to determine if the compounds were active or not active. It merely gave us information to focus on 17 compounds which were most promising in this initial screen. These compounds were then tested in an assay to determine whether they were active.

We have rewritten the Methods section as follows (line 221):

*“The 71 additional approved drugs were first prioritised by measuring their activity in an indicative screen with four adult worms in four individual wells: one at 100 µM, two at 50 µM, and one at 20 µM. This did not allow us to determine activity/non-activity of these compounds but rather enabled us to focus our investigation on compounds of interest. Seventeen compounds were selected and were tested at 100 µM and 50 µM for 24 h, with six replicates each.”*

In mice's in vivo drug screening, the authors inoculated each mouse with 20 embryonated eggs. After 35 days, they collected fecal material and generated fecal smears to check for the presence of T. muris eggs. It needs to be clarified here how the smears were prepared.

We added some sentences to the Methods on how the faecal smears were carried out (line 244):

“*Briefly, each mouse was temporarily placed in a beaker and a faecal sample collected using forceps. Half of the faecal pellet was placed on a glass slide,* ***~****50 µL of UltraPure water added and the pellet gently squashed with a second slide. The sample was then observed under the microscope (X40 objective) and classified as egg-positive or egg-negative*.”

The standard practice in the field is to use a fecal egg counting method to count the number of eggs per gram of feces. Also, more than qualitative checking of the presence of T. muris eggs in infected mice is needed for well-designed in vivo studies. A better practice is to count the number of eggs per gram of feces, then group animals into experimental groups with similar averages of the pre-treatment fecal egg count.

In our case the purpose of the faecal smear was simply to confirm the mouse infection before starting the drug treatment, as in the drug screen protocol of Keiser *et al* 2016 [PMID:27363703]. The idea of grouping animals into experimental groups with similar faecal egg count (so similar worm burden) is a nice idea, and would have been interesting to do, but unfortunately we would not have had access to enough mice to do this for each drug tested, for reasons of ethics and cost.

Mice were treated for three days starting on day 36 p.i., and animals were euthanized on day 44 p.i.  to count the worm burdens.  The authors didn’t report any post-treatment fecal egg count, a very important readout in the parasitic nematode in vivo studies. Especially in early investigation phases, candidate drugs may fail to induce worm expulsion but are effective enough to reduce egg-laying capacity significantly, an activity that could be improved.
Because our study aimed at finding approved human drugs to ‘repurpose’ as novel anti-*Trichuris* drugs, we wanted to find drugs that could reduce the worm burden as least as much as the current treatment (mebendazole), and considered that a drug that reduced egg-laying but not worm burden was unlikely to be suitable for repurposing.

Results
The result section is easy to read and straightforward, and the figures and tables are well-designed and easy to follow.  However, some issues need to be addressed here.
This header, “Pimozide was the most efficacious drug tested against T. muris in mice,” is inaccurate and may be misleading, as a 19% reduction in motility is not nearly considered the most efficacious nor considered active at all.

We thank the reviewer for this point. We have changed this heading in the Results section to be *‘Except for mebendazole, drugs were not effective against T. muris in mice’* (line 401).

In Figure 2 (E), the first figure, “Astemizole EC50 value needs to be corrected as it now reads 14 ± 48µM.

We thank the reviewer for pointing out the large error estimate on this EC_50_. This is because the concentration series we used did not in this case permit observation of an intermediate/partial effect of the drug, causing uncertainty in the EC_50_ fit. We have therefore revised Figure 2(E) to show that the astemizole EC_50_ is an estimate and added text to the figure legend (line 296) to explain the uncertainty of this value:

*“EC50 values are indicated ± the standard deviation. For astemizole, there was large uncertainty in the calculated EC50 value, so it is shown as an estimate.”*

In Figure 4, the data show that many individual mice had a worm burden above 20 worms, even though these mice were inoculated with only 20 embryonated eggs. The authors must comment on discrepancies between the results and methods reporting the in vivo studies in mice sections.

We thank the reviewer for pointing this out. We have changed the Methods to say that we infected each mouse with approximately 20-30 eggs (line 241).

Discussions
the discussion is interesting, convincing, and makes sense. In the section discussing the overlap between C. elegans and whipworms, the authors referred to the suitability of C. elegans as a surrogate model for whipworms. However, their data doesn’t support that claim, as only two of the 50 compounds active against whipworms overlap with C. elegans. A similar finding was reported recently when testing the quality of C. elegans in predicting antiparasitic nematode activity and found that C. elegans is prone to a higher rate of false negatives. I suggest that the authors reference opinions that argue against the suitability of C. elegans in modeling parasitic nematodes.

We thank the reviewer for these comments. We have now altered the text in the Discussion to say (line 444):

*“C. elegans is often used as a proxy for whipworms and other parasitic nematodes in drug screens (Kwok et al. 2006; Burns et al. 2015), and functional assays in C. elegans have aided the development of new anthelmintics such as the amino-acetonitrile derivative monepantel (Kaminsky et al. 2008). However, for some screening libraries, there has been relatively low overlap reported between hits in* C. elegans *and a particular parasitic nematode (e.g. for the human hookworm* Ancylostoma ceylanicum in *Elfawal et al 2019 PMID:31451730). Indeed, when our drug library was screened against* C. elegans*, only two of the top 50 hits against whipworm were also hits against* C. elegans*.”*

The authors greatly discussed the reasons behind the discrepancy between ex vivo potency and in vivo activity. They offered exciting tips for dealing with this critical problem challenging the anthelmintic drug discovery efforts.
We again thank the reviewer for these comments.
